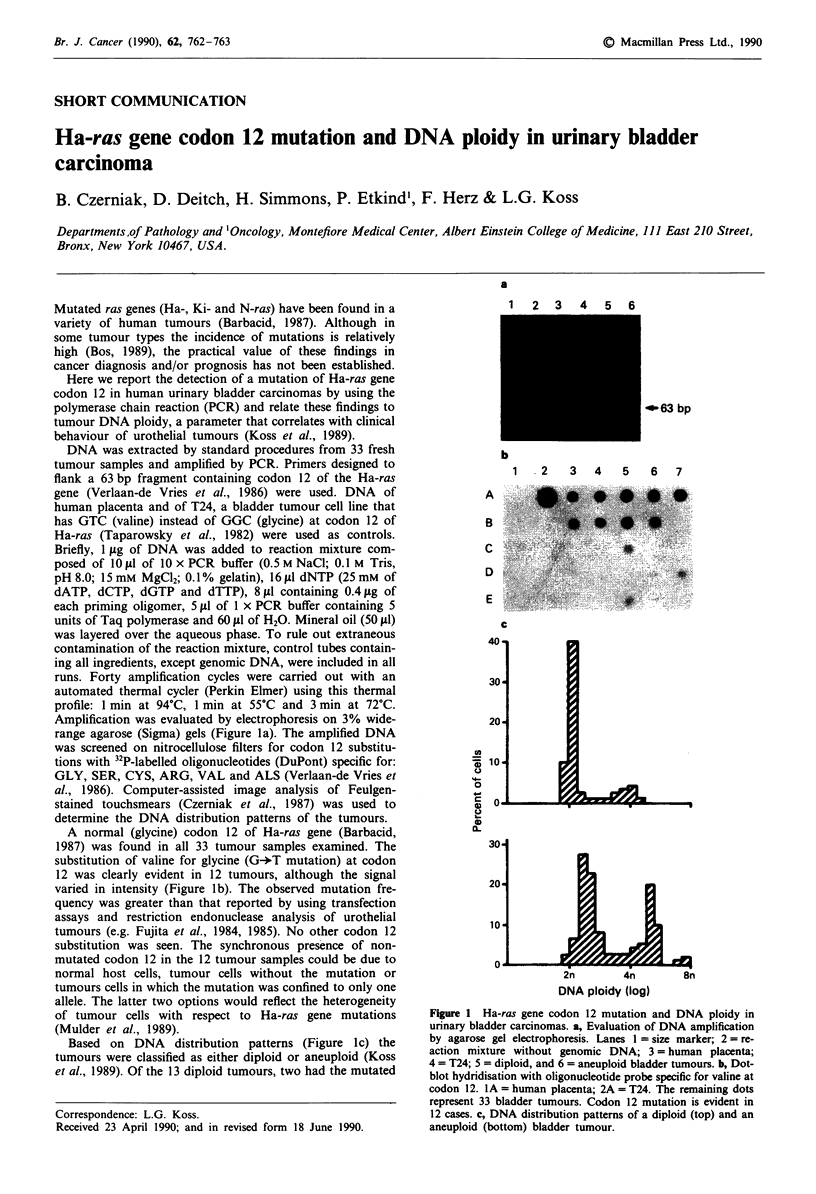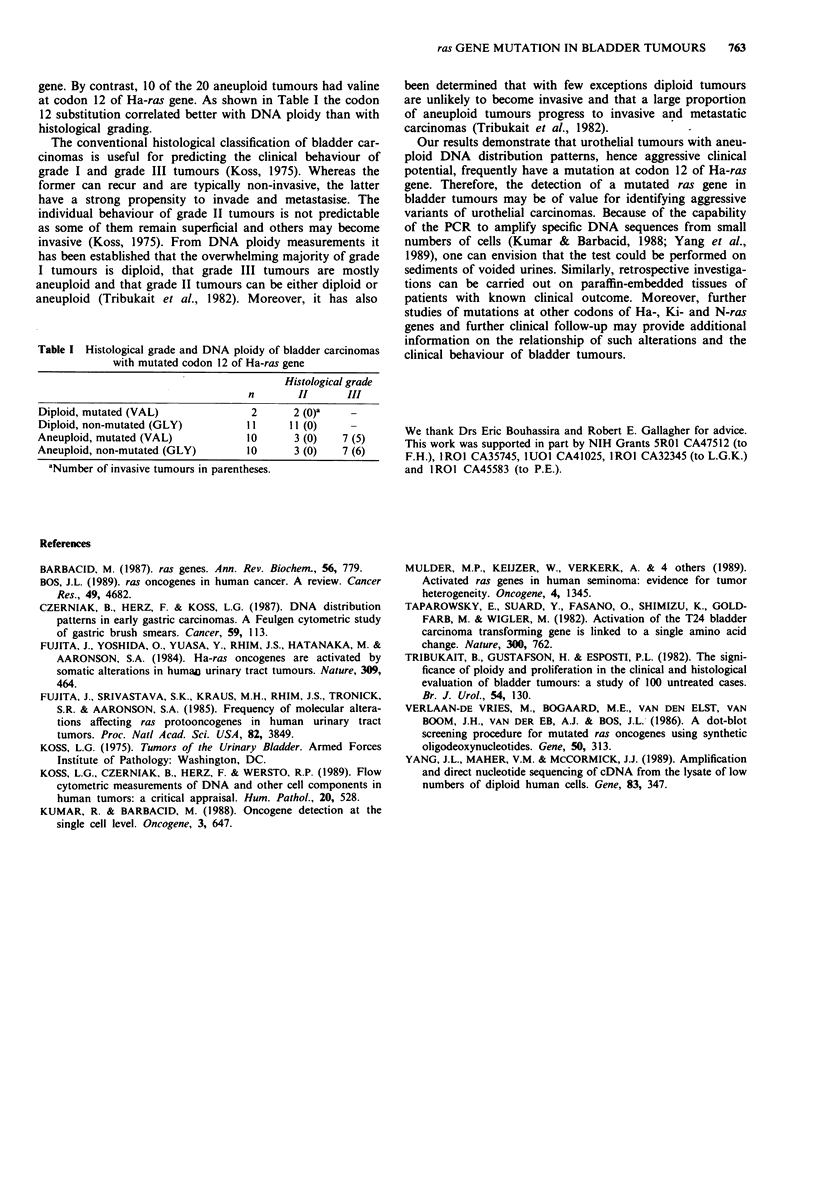# Ha-ras gene codon 12 mutation and DNA ploidy in urinary bladder carcinoma.

**DOI:** 10.1038/bjc.1990.374

**Published:** 1990-11

**Authors:** B. Czerniak, D. Deitch, H. Simmons, P. Etkind, F. Herz, L. G. Koss

**Affiliations:** Department of Pathology, Montefiore Medical Center, Albert Einstein College of Medicine, Bronx, New York 10467.

## Abstract

**Images:**


Br. J. Cancer (1990), 62, 762-763 © Macmillan Press Ltd., 1990

SHORT COMMUNICATION

### Ha-*ras* gene codon 12 mutation and DNA ploidy in urinary bladder carcinoma

B. Czerniak, D. Deitch, H. Simmons, P. Etkind[1], F. Herz & L.G. Koss

*Departments of Pathology and [1]Oncology, Montefiore Medical Center, Albert Einstein College of Medicine, 111 East 210 Street, Bronx, New York 10467, USA.*

Mutated *ras* genes (Ha-, Ki- and N-*ras*) have been found in a variety of human tumours (Barbacid, 1987). Although in some tumour types the incidence of mutations is relatively high (Bos, 1989), the practical value of these findings in cancer diagnosis and/or prognosis has not been established.

Here we report the detection of a mutation of Ha-*ras* gene codon 12 in human urinary bladder carcinomas by using the polymerase chain reaction (PCR) and relate these findings to tumour DNA ploidy, a parameter that correlates with clinical behaviour of urothelial tumours (Koss *et al.*, 1989).

DNA was extracted by standard procedures from 33 fresh tumour samples and amplified by PCR. Primers designed to flank a 63 bp fragment containing codon 12 of the Ha-*ras* gene (Verlaan-de Vries *et al.*, 1986) were used. DNA of human placenta and of T24, a bladder tumour cell line that has GTC (valine) instead of GGC (glycine) at codon 12 of Ha-*ras* (Taparowsky *et al.*, 1982) were used as controls. Briefly, 1 µg of DNA was added to reaction mixture composed of 10 µl of 10 × PCR buffer (0.5 M NaCl; 0.1 M Tris, pH 8.0; 15 mM $MgCl_2$; 0.1% gelatin), 16 µl dNTP (25 mM of dATP, dCTP, dGTP and dTTP), 8 µl containing 0.4 µg of each priming oligomer, 5 µl of 1 × PCR buffer containing 5 units of Taq polymerase and 60 µl of $H_2O$. Mineral oil (50 µl) was layered over the aqueous phase. To rule out extraneous contamination of the reaction mixture, control tubes containing all ingredients, except genomic DNA, were included in all runs. Forty amplification cycles were carried out with an automated thermal cycler (Perkin Elmer) using this thermal profile: 1 min at 94°C, 1 min at 55°C and 3 min at 72°C. Amplification was evaluated by electrophoresis on 3% wide-range agarose (Sigma) gels (Figure 1a). The amplified DNA was screened on nitrocellulose filters for codon 12 substitutions with $^{32}P$-labelled oligonucleotides (DuPont) specific for: GLY, SER, CYS, ARG, VAL and ALS (Verlaan-de Vries *et al.*, 1986). Computer-assisted image analysis of Feulgen-stained touchsmears (Czerniak *et al.*, 1987) was used to determine the DNA distribution patterns of the tumours.

A normal (glycine) codon 12 of Ha-*ras* gene (Barbacid, 1987) was found in all 33 tumour samples examined. The substitution of valine for glycine (G→T mutation) at codon 12 was clearly evident in 12 tumours, although the signal varied in intensity (Figure 1b). The observed mutation frequency was greater than that reported by using transfection assays and restriction endonuclease analysis of urothelial tumours (e.g. Fujita *et al.*, 1984, 1985). No other codon 12 substitution was seen. The synchronous presence of non-mutated codon 12 in the 12 tumour samples could be due to normal host cells, tumour cells without the mutation or tumours cells in which the mutation was confined to only one allele. The latter two options would reflect the heterogeneity of tumour cells with respect to Ha-*ras* gene mutations (Mulder *et al.*, 1989).

Based on DNA distribution patterns (Figure 1c) the tumours were classified as either diploid or aneuploid (Koss *et al.*, 1989). Of the 13 diploid tumours, two had the mutated

**Figure 1** Ha-*ras* gene codon 12 mutation and DNA ploidy in urinary bladder carcinomas. **a**, Evaluation of DNA amplification by agarose gel electrophoresis. Lanes 1 = size marker; 2 = reaction mixture without genomic DNA; 3 = human placenta; 4 = T24; 5 = diploid, and 6 = aneuploid bladder tumours. **b**, Dot-blot hydridisation with oligonucleotide probe specific for valine at codon 12. 1A = human placenta; 2A = T24. The remaining dots represent 33 bladder tumours. Codon 12 mutation is evident in 12 cases. **c**, DNA distribution patterns of a diploid (top) and an aneuploid (bottom) bladder tumour.

Correspondence: L.G. Koss.
Received 23 April 1990; and in revised form 18 June 1990.

gene. By contrast, 10 of the 20 aneuploid tumours had valine at codon 12 of Ha-*ras* gene. As shown in Table I the codon 12 substitution correlated better with DNA ploidy than with histological grading.

The conventional histological classification of bladder carcinomas is useful for predicting the clinical behaviour of grade I and grade III tumours (Koss, 1975). Whereas the former can recur and are typically non-invasive, the latter have a strong propensity to invade and metastasise. The individual behaviour of grade II tumours is not predictable as some of them remain superficial and others may become invasive (Koss, 1975). From DNA ploidy measurements it has been established that the overwhelming majority of grade I tumours is diploid, that grade III tumours are mostly aneuploid and that grade II tumours can be either diploid or aneuploid (Tribukait *et al.*, 1982). Moreover, it has also been determined that with few exceptions diploid tumours are unlikely to become invasive and that a large proportion of aneuploid tumours progress to invasive and metastatic carcinomas (Tribukait *et al.*, 1982).

Our results demonstrate that urothelial tumours with aneuploid DNA distribution patterns, hence aggressive clinical potential, frequently have a mutation at codon 12 of Ha-*ras* gene. Therefore, the detection of a mutated *ras* gene in bladder tumours may be of value for identifying aggressive variants of urothelial carcinomas. Because of the capability of the PCR to amplify specific DNA sequences from small numbers of cells (Kumar & Barbacid, 1988; Yang *et al.*, 1989), one can envision that the test could be performed on sediments of voided urines. Similarly, retrospective investigations can be carried out on paraffin-embedded tissues of patients with known clinical outcome. Moreover, further studies of mutations at other codons of Ha-, Ki- and N-*ras* genes and further clinical follow-up may provide additional information on the relationship of such alterations and the clinical behaviour of bladder tumours.

**Table I** Histological grade and DNA ploidy of bladder carcinomas with mutated codon 12 of Ha-*ras* gene

| | | *Histological grade* | |
|---|---|---|---|
| | *n* | *II* | *III* |
| Diploid, mutated (VAL) | 2 | 2 (0)[a] | – |
| Diploid, non-mutated (GLY) | 11 | 11 (0) | – |
| Aneuploid, mutated (VAL) | 10 | 3 (0) | 7 (5) |
| Aneuploid, non-mutated (GLY) | 10 | 3 (0) | 7 (6) |

[a]Number of invasive tumours in parentheses.

We thank Drs Eric Bouhassira and Robert E. Gallagher for advice. This work was supported in part by NIH Grants 5R01 CA47512 (to F.H.), 1RO1 CA35745, 1UO1 CA41025, 1RO1 CA32345 (to L.G.K.) and 1RO1 CA45583 (to P.E.).

### References

BARBACID, M. (1987). *ras* genes. *Ann. Rev. Biochem.*, **56**, 779.

BOS, J.L. (1989). *ras* oncogenes in human cancer. A review. *Cancer Res.*, **49**, 4682.

CZERNIAK, B., HERZ, F. & KOSS, L.G. (1987). DNA distribution patterns in early gastric carcinomas. A Feulgen cytometric study of gastric brush smears. *Cancer*, **59**, 113.

FUJITA, J., YOSHIDA, O., YUASA, Y., RHIM, J.S., HATANAKA, M. & AARONSON, S.A. (1984). Ha-*ras* oncogenes are activated by somatic alterations in human urinary tract tumours. *Nature*, **309**, 464.

FUJITA, J., SRIVASTAVA, S.K., KRAUS, M.H., RHIM, J.S., TRONICK, S.R. & AARONSON, S.A. (1985). Frequency of molecular alterations affecting *ras* protooncogenes in human urinary tract tumors. *Proc. Natl Acad. Sci. USA*, **82**, 3849.

KOSS, L.G. (1975). *Tumors of the Urinary Bladder*. Armed Forces Institute of Pathology: Washington, DC.

KOSS, L.G., CZERNIAK, B., HERZ, F. & WERSTO, R.P. (1989). Flow cytometric measurements of DNA and other cell components in human tumors: a critical appraisal. *Hum. Pathol.*, **20**, 528.

KUMAR, R. & BARBACID, M. (1988). Oncogene detection at the single cell level. *Oncogene*, **3**, 647.

MULDER, M.P., KEIJZER, W., VERKERK, A. & 4 others (1989). Activated *ras* genes in human seminoma: evidence for tumor heterogeneity. *Oncogene*, **4**, 1345.

TAPAROWSKY, E., SUARD, Y., FASANO, O., SHIMIZU, K., GOLDFARB, M. & WIGLER, M. (1982). Activation of the T24 bladder carcinoma transforming gene is linked to a single amino acid change. *Nature*, **300**, 762.

TRIBUKAIT, B., GUSTAFSON, H. & ESPOSTI, P.L. (1982). The significance of ploidy and proliferation in the clinical and histological evaluation of bladder tumours: a study of 100 untreated cases. *Br. J. Urol.*, **54**, 130.

VERLAAN-DE VRIES, M., BOGAARD, M.E., VAN DEN ELST, VAN BOOM, J.H., VAN DER EB, A.J. & BOS, J.L. (1986). A dot-blot screening procedure for mutated *ras* oncogenes using synthetic oligodeoxynucleotides. *Gene*, **50**, 313.

YANG, J.L., MAHER, V.M. & MCCORMICK, J.J. (1989). Amplification and direct nucleotide sequencing of cDNA from the lysate of low numbers of diploid human cells. *Gene*, **83**, 347.